# Supplementary material for: Stomata-targeted nanocarriers enhance plant defense against pathogen colonization
Source: Nat Commun. 2025 May 23;16:4816. doi: 10.1038/s41467-025-60112-w (PMC12102249; doi:10.1038/s41467-025-60112-w)
Supplement: Supplementary file 1 — Supplementary Information [file 41467_2025_60112_MOESM1_ESM.docx]

Supplementary Information

**Stomata-Targeted Nanocarriers Enhance Plant Defense Against Pathogen Colonization**

Suppanat Puangpathumanond,^1^ Heng Li Chee,^2^ Cansu Sevencan,^1^ Xin Yang,^3^ On Sun Lau,^3,4^ Tedrick Thomas Salim Lew^1,4*^

^1^ Department of Chemical and Biomolecular Engineering, National University of Singapore, Singapore, Singapore.

^2^ Institute of Materials Research and Engineering, Agency of Science, Technology and Research, Singapore, Singapore.

^3^ Department of Biological Sciences, National University of Singapore, Singapore, Singapore.

^4^ Research Centre on Sustainable Urban Farming, National University of Singapore, Singapore, Singapore.

*Corresponding author. Email: [tedrick@nus.edu.sg](mailto:tedrick@nus.edu.sg)

**
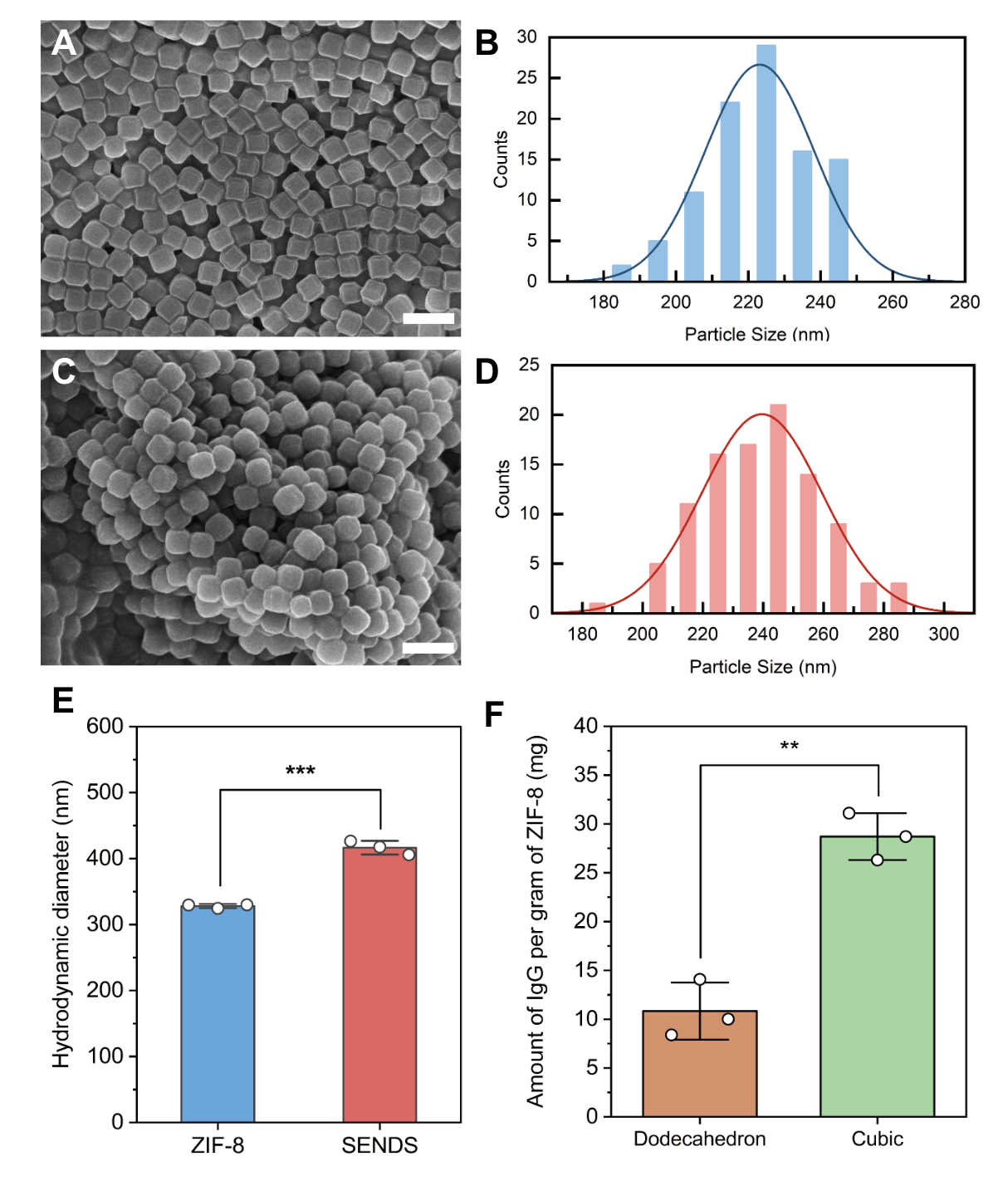
**

**Fig. S1. Characterization of physical properties of SENDS.** Wide-view SEM micrographs of (**A**) ZIF-8 and (**C**) SENDS, highlighting monodispersity and consistent morphology. Scale bar, 500 nm. Size distributions of (**B**) ZIF-8 and (**D**) SENDS. **(E)** Increased hydrodynamic diameter of SENDS following biomolecule functionalization. **(F)** A comparison of ZIF-8 IgG binding capacity for dodecahedral and cubic morphologies. Data are presented as mean ± SD. (n=3 technical replicates). Statistical differences were calculated using two-sample t-test. **P<0.01, ***P<0.001.


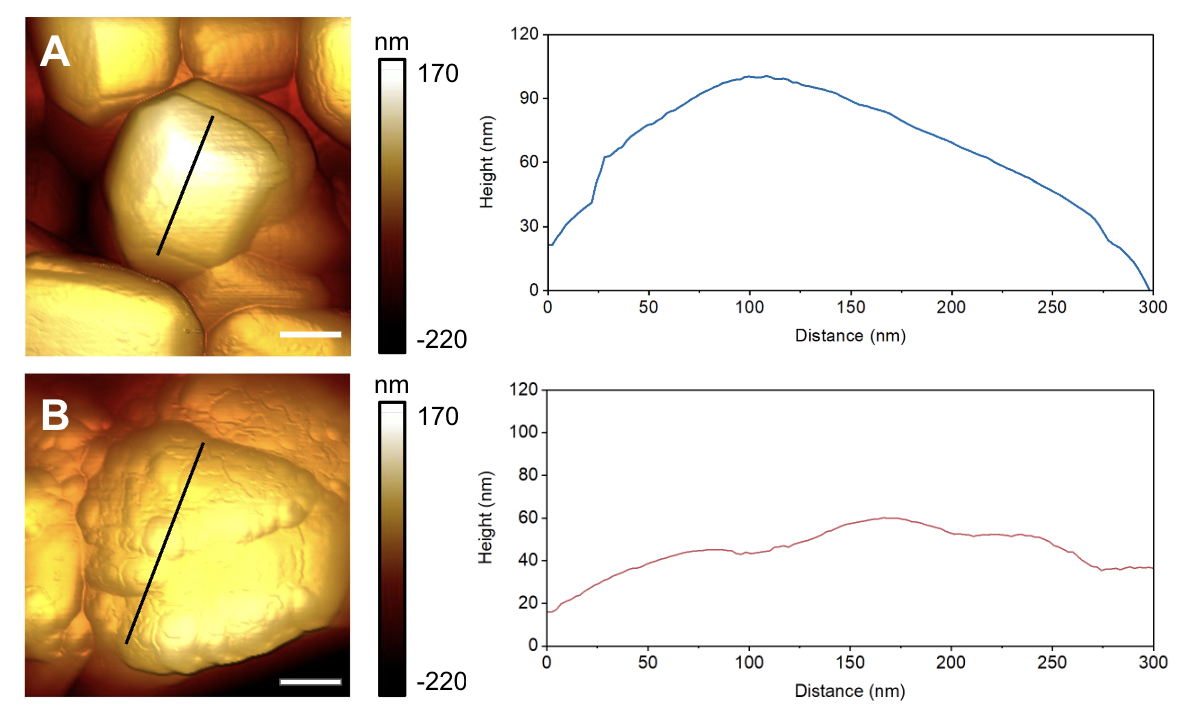


**Fig. S2: Surface roughness analysis of ZIF-8 and SENDS.** AFM images and corresponding line profiles of (**A**) ZIF-8 and (**B**) SENDS demonstrate increased surface roughness following functionalization. Scale bar, 100 nm.


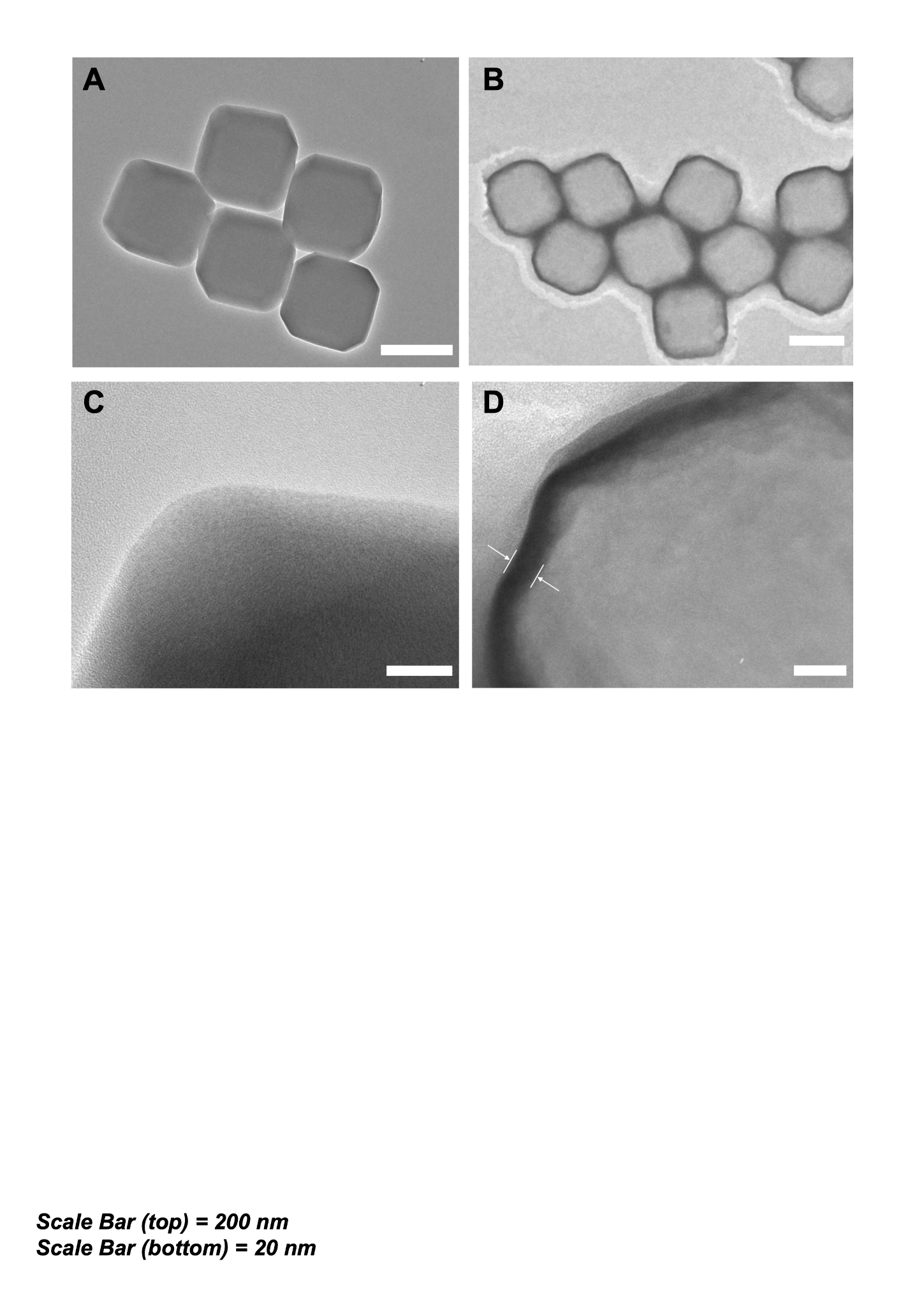


**Fig. S3.** **TEM micrographs of stained ZIF-8 and SENDS to highlight the presence of the functionalized biomolecule layer on SENDS.** (**A)** ZIF-8 and (**B**) SENDS; scale bar, 200 nm. (**C**) ZIF-8 and (**D**) SENDS; scale bar, 20 nm. Thickness of the biomolecule layer on the surface of SENDS is estimated to be approximately 15 nm.


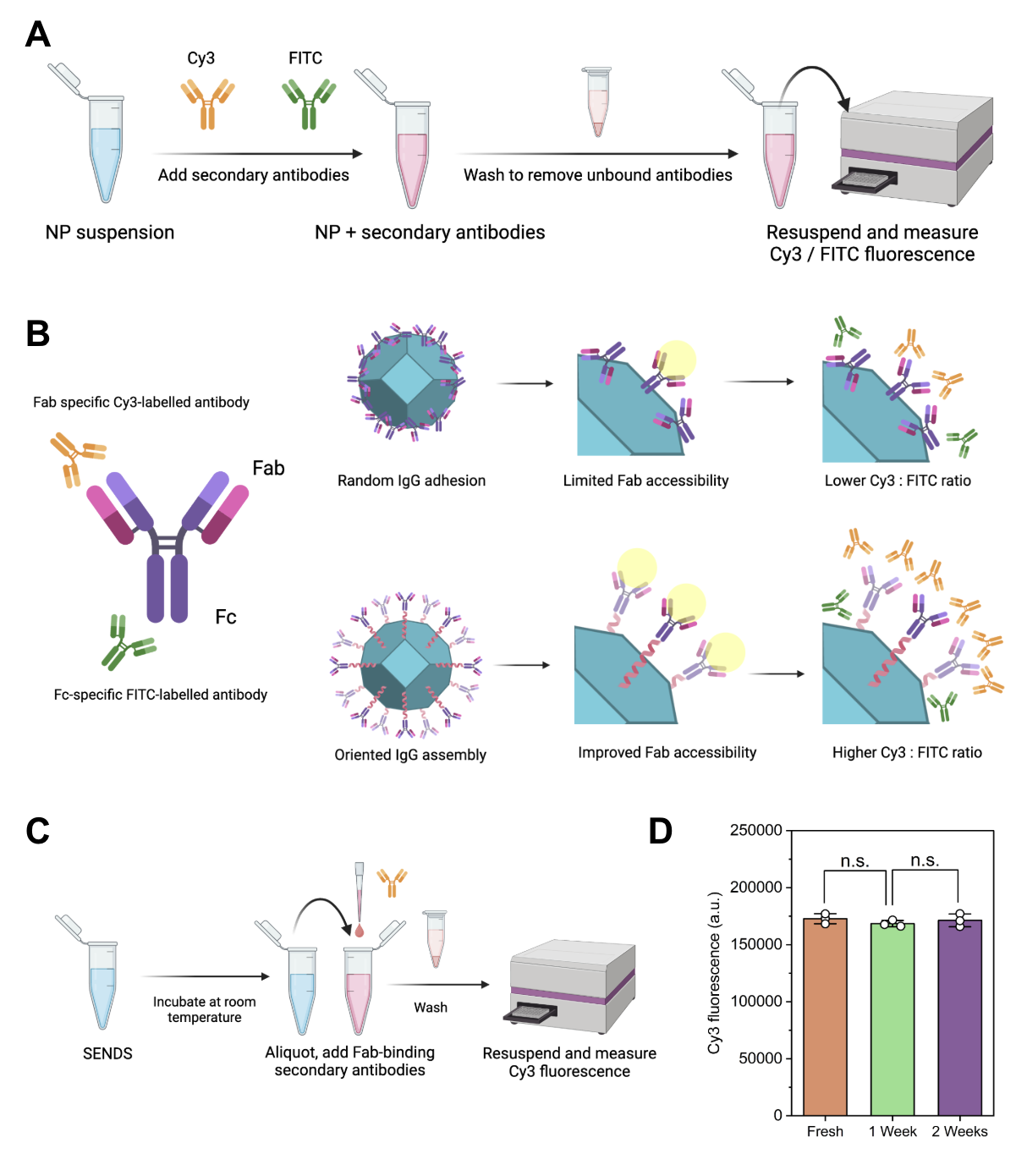


**Fig. S4. LM6 IgG orientation and SENDS stability studies.** (**A**) Schematic detailing experimental procedure to assess IgG orientation on the nanoparticle surface. (**B**) Schematic describing how Cy3: FITC ratios can be used to assess the accessibility of Fab and Fc regions. (**C**) Schematic detailing stability study protocol. (**D**) Minimal changes in Cy3 secondary antibody fluorescence over time suggest that the biorecognition function of the LM6 IgG adhered to SENDS remains intact during 2-week storage under ambient conditions. Data are presented as mean ± SD (n=3). Statistical differences were calculated using ANOVA with Tukey’s post-hoc test. Schematics created in BioRender. Lew, T.T.S. (2025) https://BioRender.com/zg9ve1d


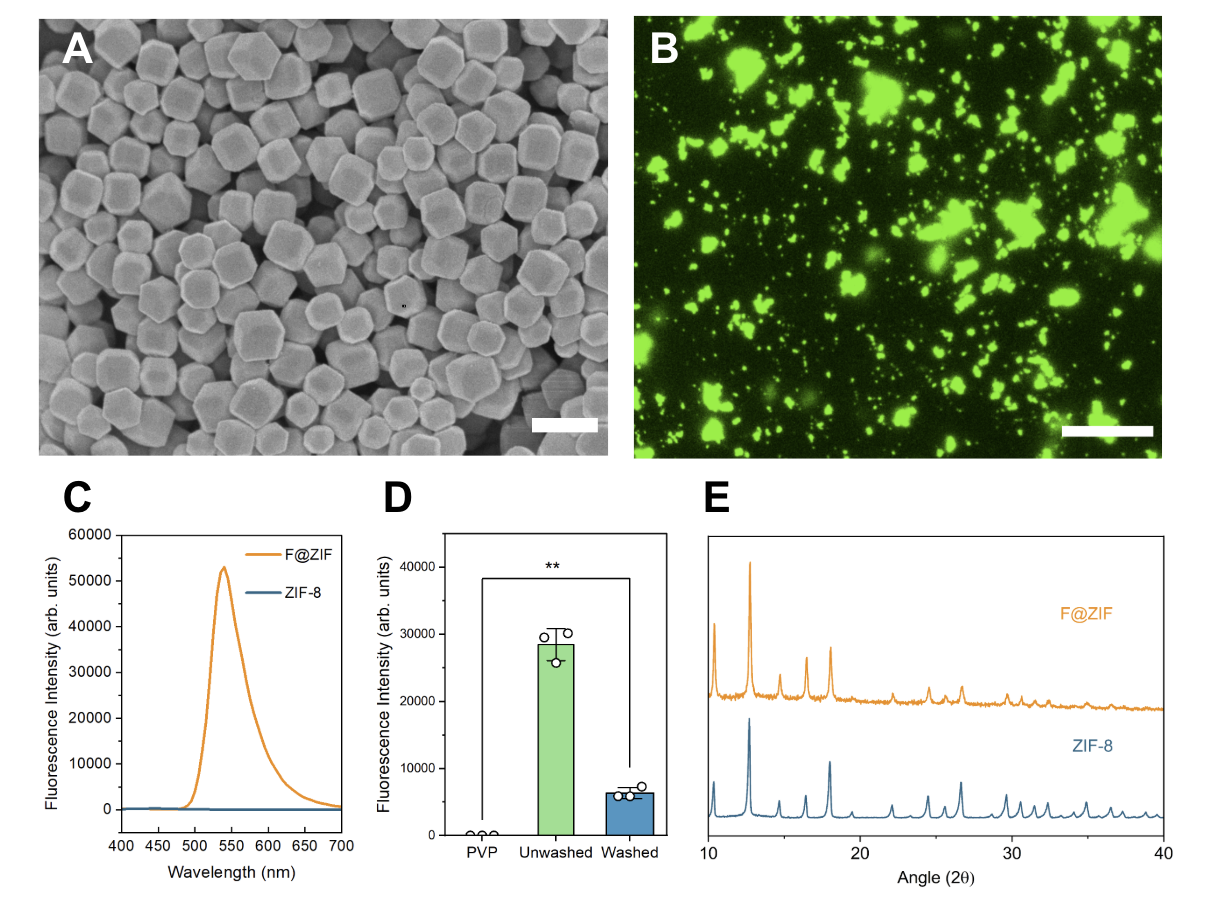


**Fig. S5.** **Characterization of F@ZIF-8.** (**A)** SEM micrograph of F@ZIF-8. Scale bar, 200 nm. (**B**) Confocal micrograph of F@ZIF-8 dispersion. Scale bar, 50 𝜇m. (**C**) Fluorescence of F@ZIF-8 in contrast to ZIF-8 under 360 nm excitation, (**D**) Fluorescence intensity of unwashed F@ZIF-8 and F@ZIF-8 washed using PVP under 360 nm excitation. Data are presented as mean ± SD (n=3 technical replicates). Statistical differences were calculated using ANOVA with Tukey’s post-hoc test. **P<0.01. (**E**) PXRD diffractograms of F@ZIF-8 and ZIF-8.


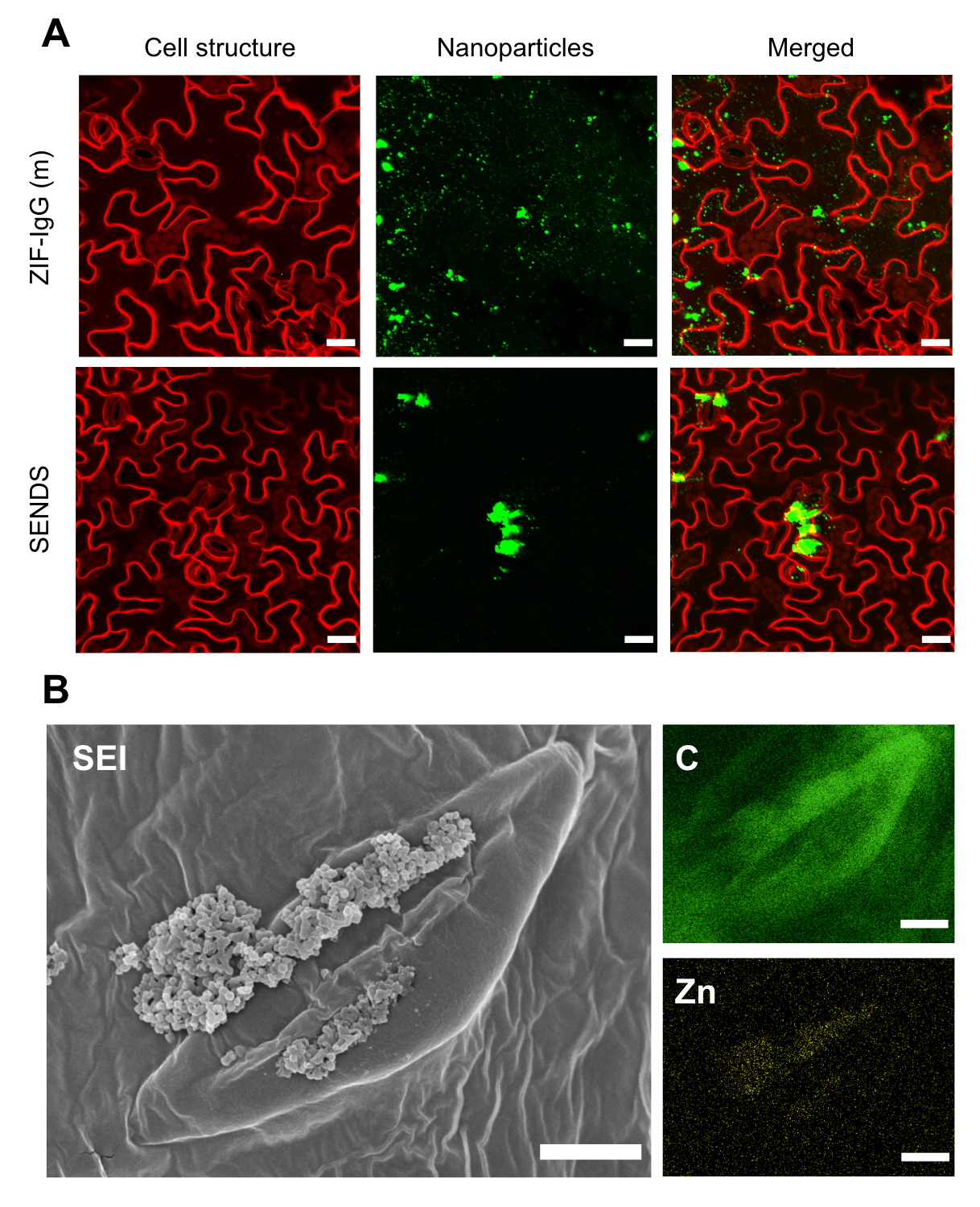


**Fig. S6.** **SENDS localization on A. thaliana.** (**A**) A comparison of NP localization between ZIF-8 functionalized with His-tagged HRP mouse monoclonal IgG and SENDS. Scale bar, 20 𝜇m (**B**) EDS mapping of stomata-localized SENDS. Scale bar, 2 𝜇m.

**
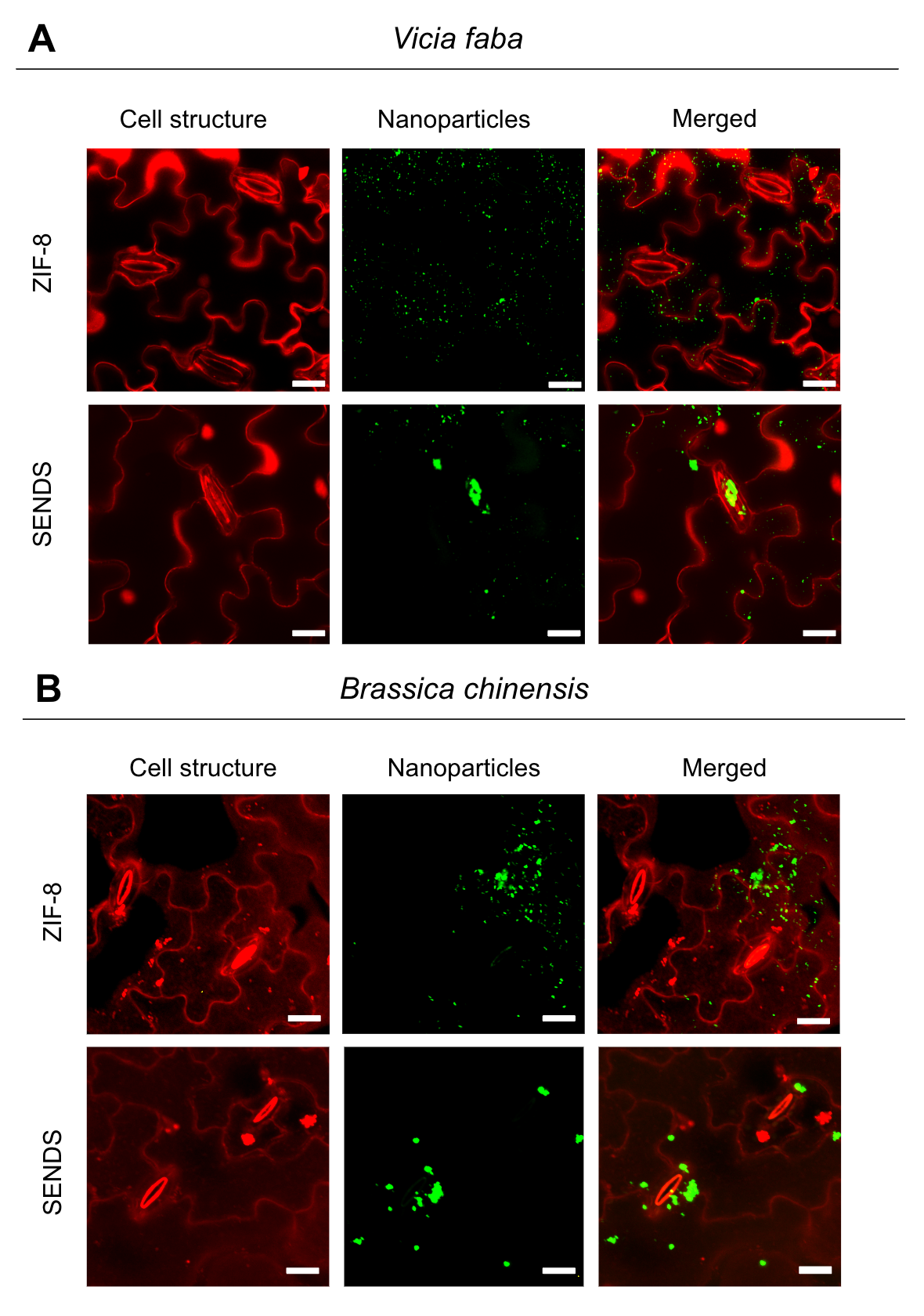
**

Fig. S7. SENDS localization in other dicot species. Confocal micrographs exhibiting localization of SENDS for (A) *V. faba* and (B) *B. chinensis*. Scale bar, 20 𝜇m.


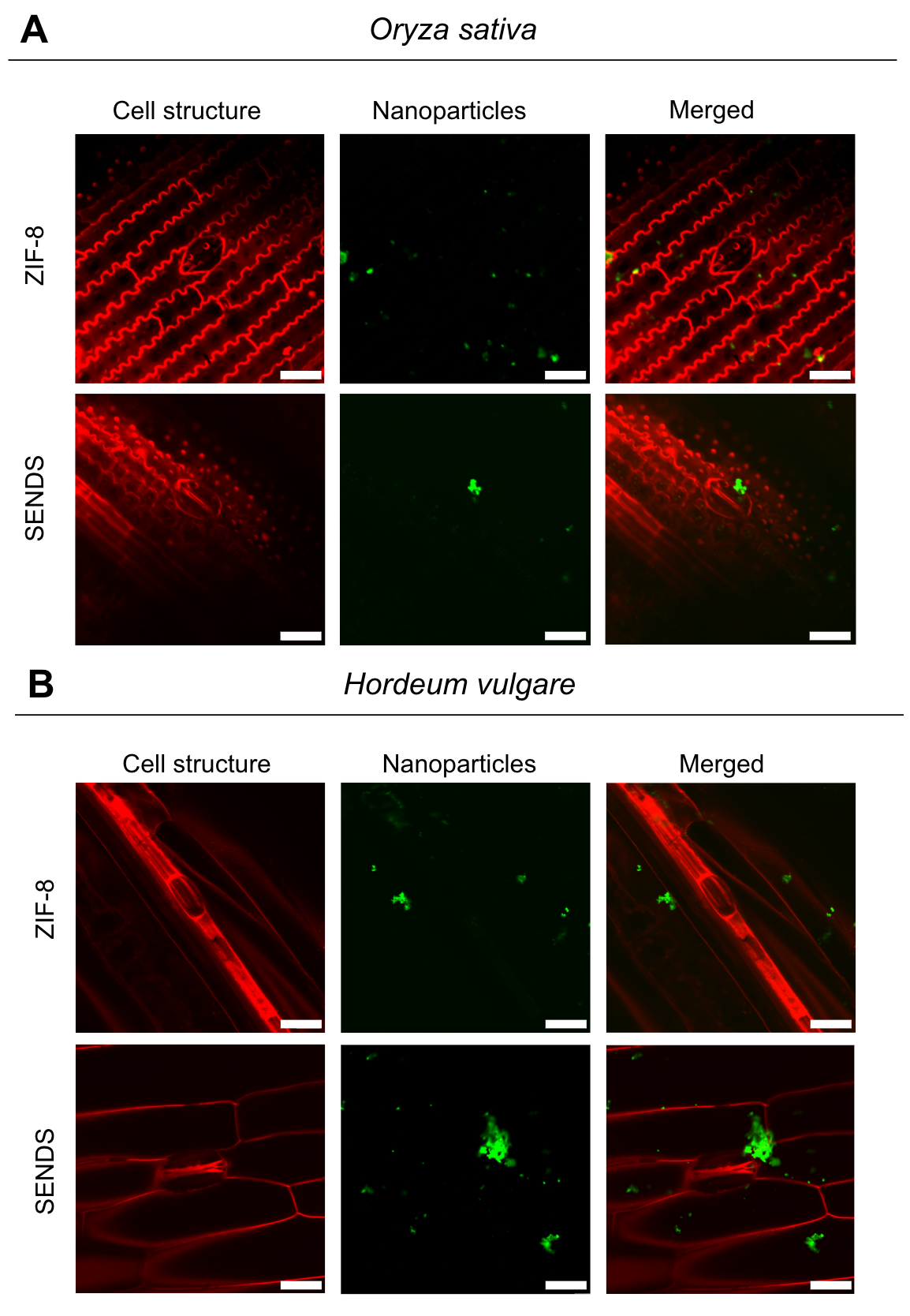


**Fig. S8. SENDS localization in monocot species.** Confocal micrographs exhibiting localization of SENDS for (**A**) *O. sativa and (***B***) H. vulgare*. Scale bar, 20 𝜇m.


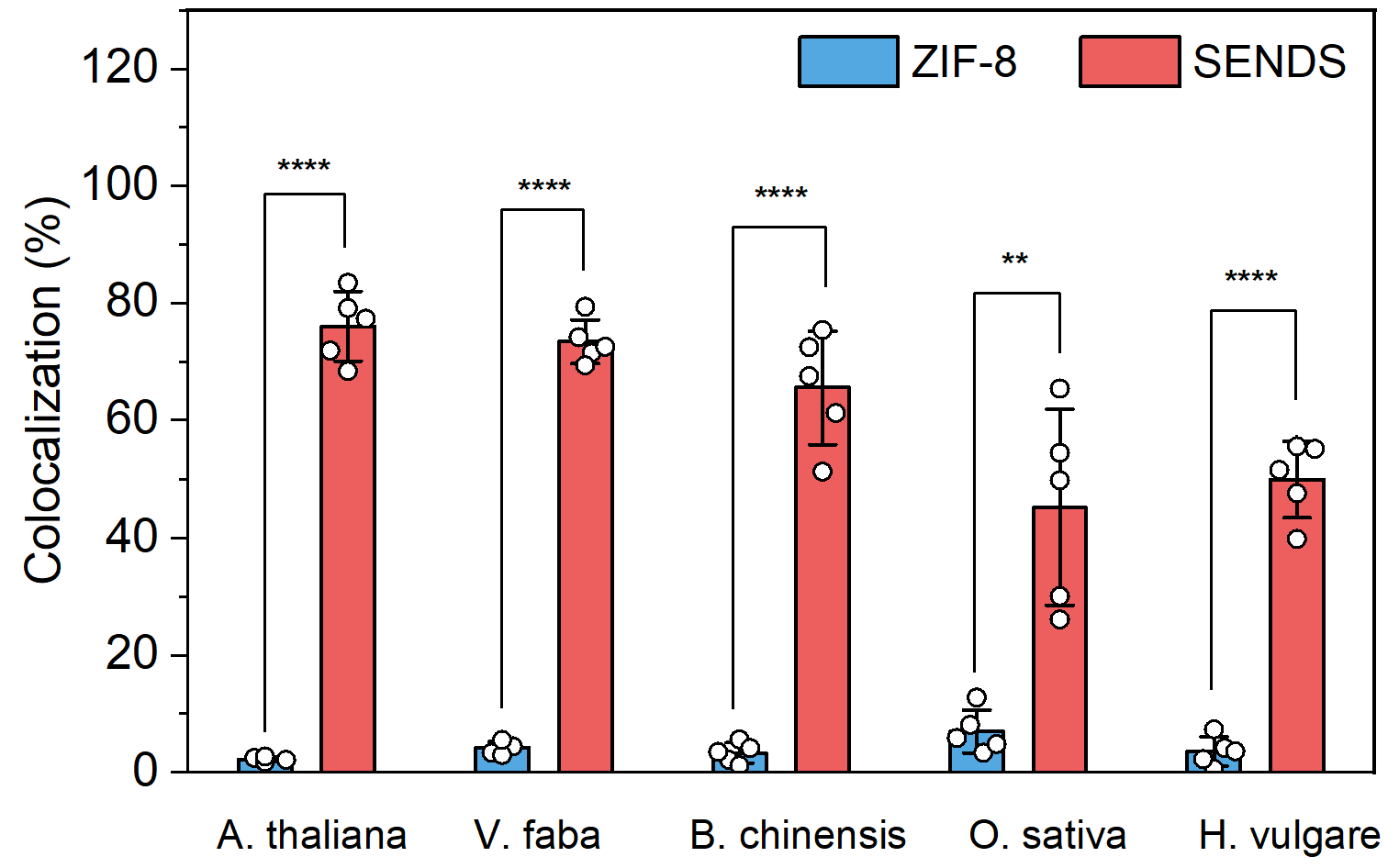


**Fig. S9. Average colocalization percentage of SENDS and ZIF-8 across all five tested plant species based on CLSM micrographs.** Data are presented as mean ± SD (n = 5 technical replicates). Statistical differences were calculated using two-sample t-test. ****P<0.0001, **P<0.01

**
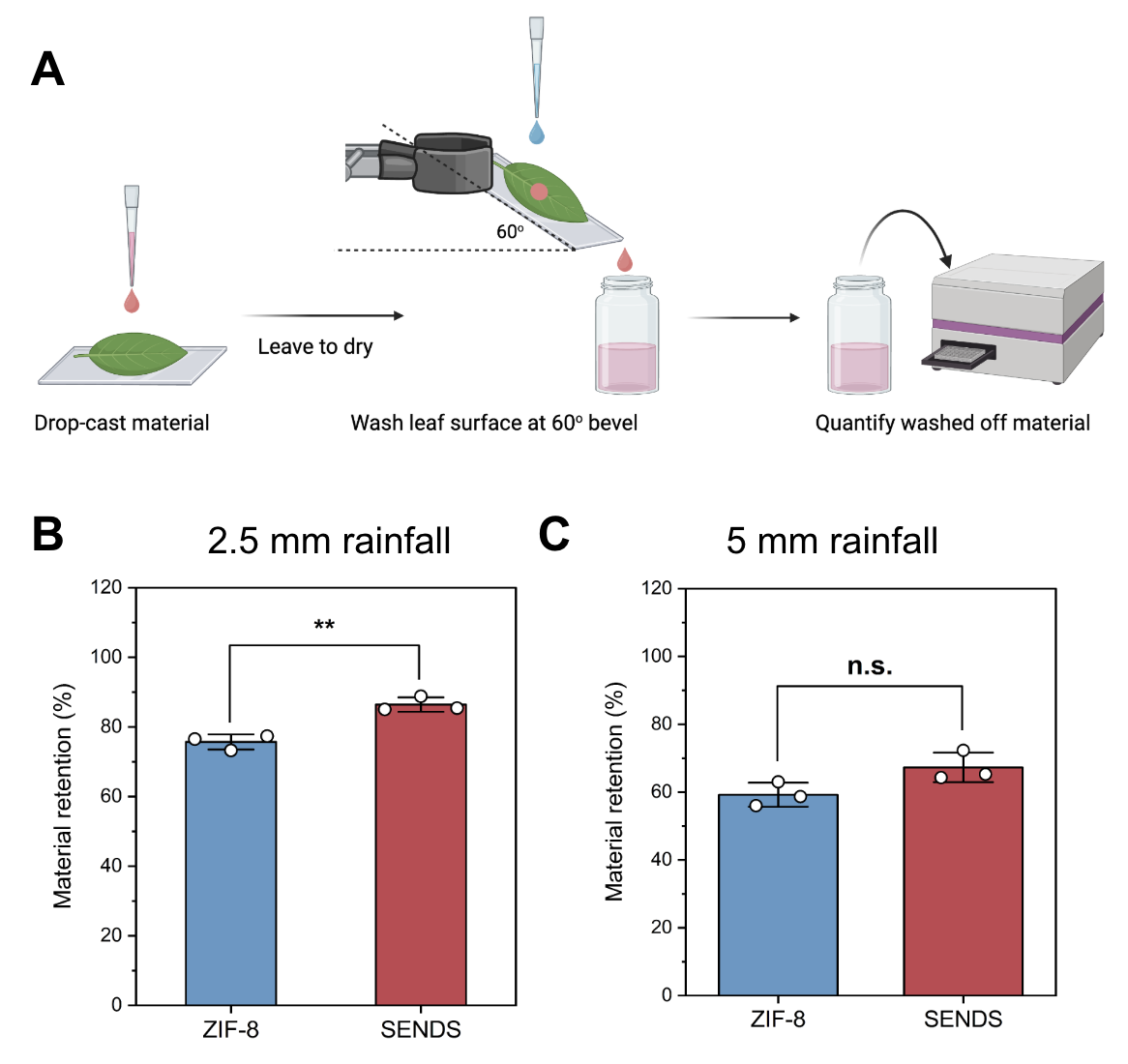
**

**Fig. S10. Rainfastness studies.** (**A**) Schematic detailing rainfastness experimental protocol. Created in BioRender. Lew, T.T.S. (2025) https://BioRender.com/r25bezm. Rainfastness of ZIF-8 and SENDS under (**B**) 2.5 mm and (**C**) 5 mm rainfall. Data are presented as mean ± SD. (n = 3 independent biological replicates). Statistical differences were calculated using two-sample t-test. **P<0.01.


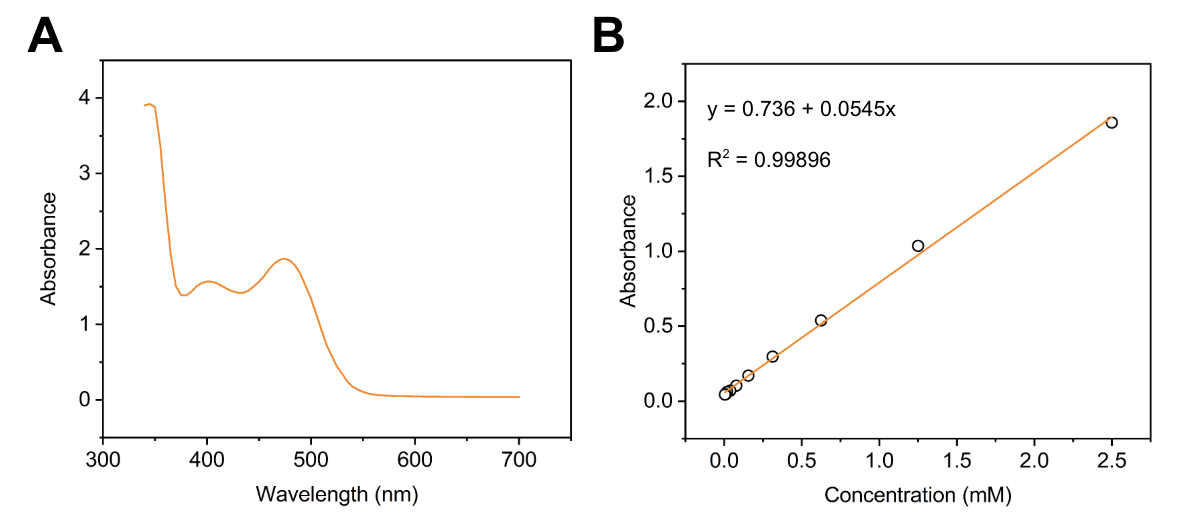


**Fig. S11.** **Determining encapsulation efficiency of SC in ZIF-8.** (**A**) Absorbance spectrum of SC, (**B**) Calibration curve for SC based on absorption maximum at 475 nm.

**
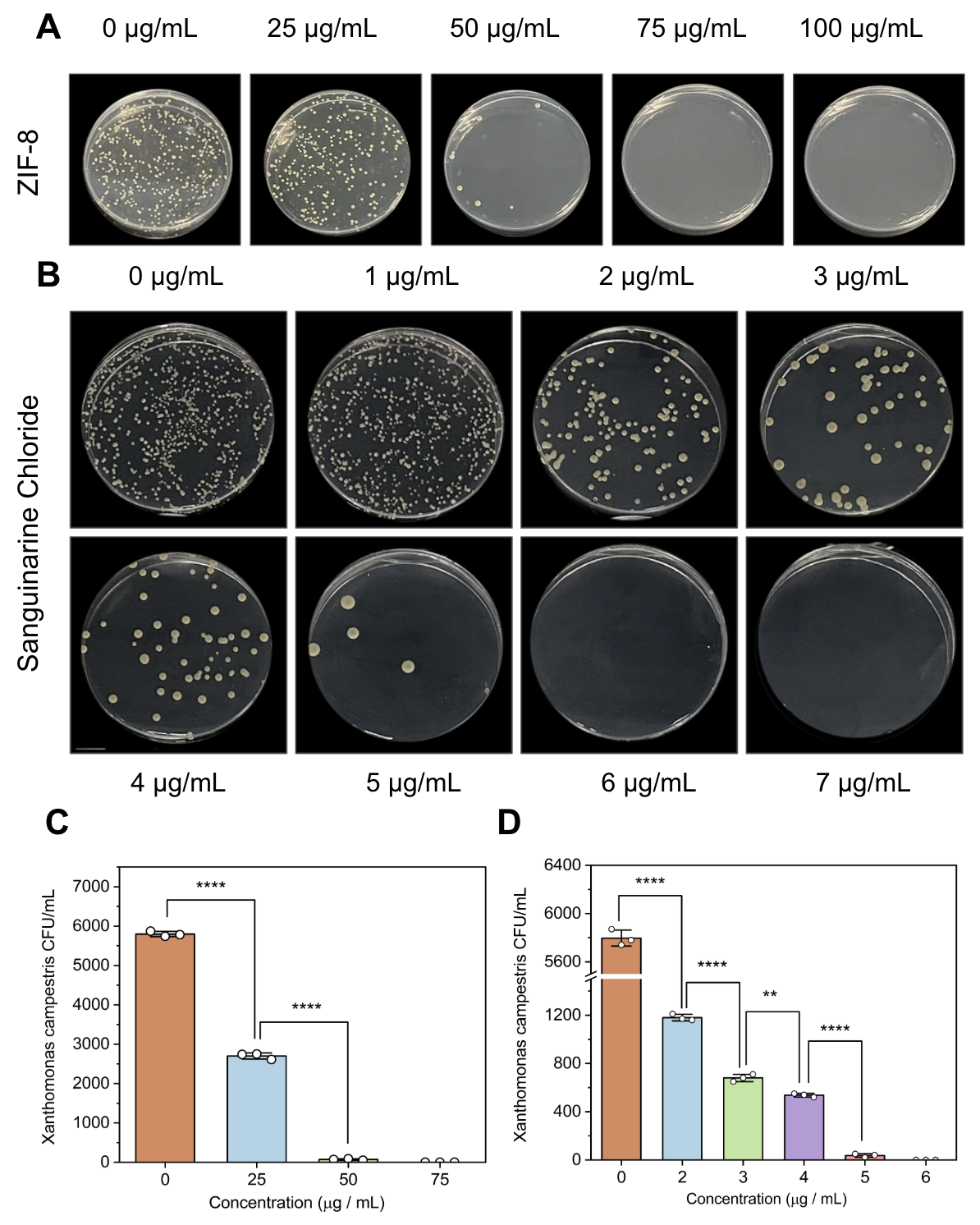
**

**Fig. S12.** **In vitro antibacterial tests.** CFU assay for various concentrations of (**A**) ZIF-8 and (**B**) sanguinarine chloride. Inhibition of (**C**) ZIF-8 and (**D**) sanguinarine chloride with increasing treatment concentrations. Data are presented as mean ± SD (n=3 independent biological replicates). Statistical differences were calculated using ANOVA with Tukey’s post-hoc test. **P<0.01, ****P<0.0001.


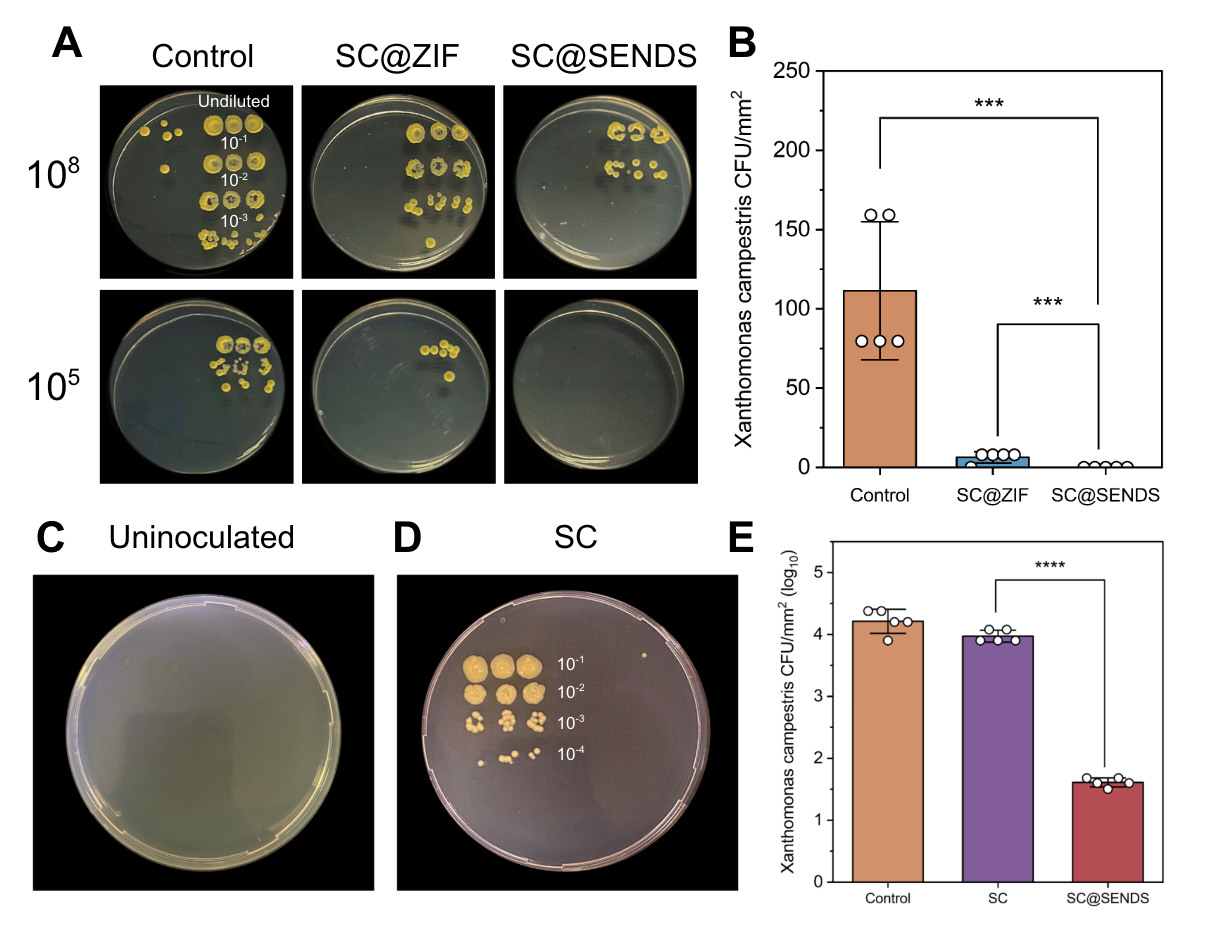


**Fig. S13.** **Quantification of bacterial load in apoplast.** (**A**) Drop plate assay for *B. chinensis* plants inoculated with 10^8^ and 10^5^ CFU/mL *X. campestris.* (**B**) Average bacterial loads of *X. campestris* in leaf apoplast of plants inoculated with 10^5^ CFU/L *X. campestris*, quantified as CFU per mm^2^. (**C**) Drop plate assay for uninoculated control showing no CFU growth. (**D**) Drop plate assay for free SC-treated B. chinensis inoculated with 10^8^ CFU/mL *X. campestris*. (**E**) Average bacterial load in leaf apoplast of free SC-treated plants in comparison to untreated and SC@SENDS treated plants. Data are presented as mean ± SD (n=5 independent biological replicates). Statistical differences were calculated using two-sample t-test. ***P<0.001, ****P<0.0001.

**
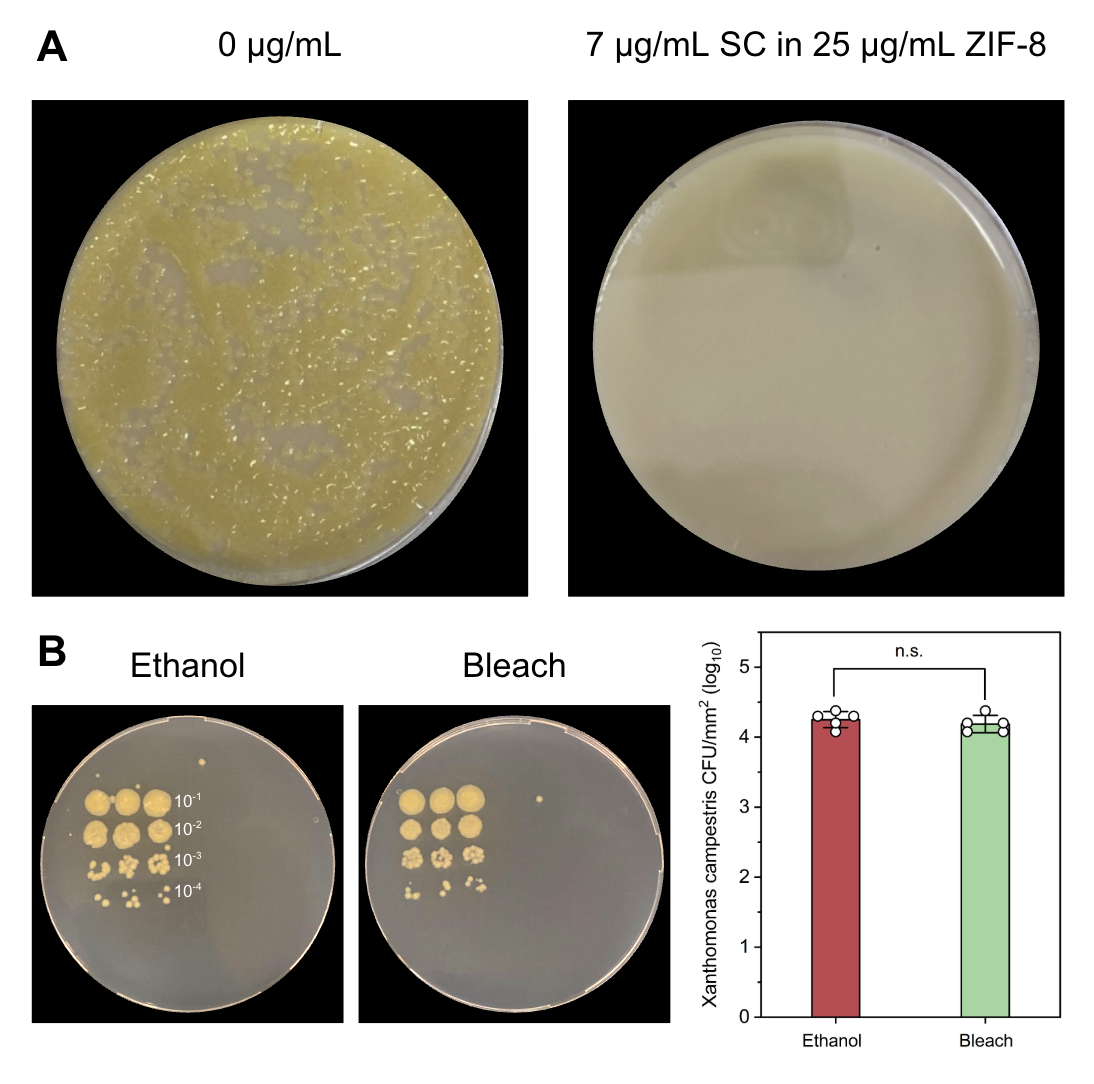
**

**Fig. S14.** **Bacteria viability evaluation under varying experimental conditions.** (**A**) CFU assay for *X. campestris* conducted at 10^6^ CFU / mL initial inoculum on YGC agar. No CFU growth was observed when incubated with 7 µg/mL SC in 25 µg/mL ZIF-8, indicating that MBC is maintained despite a higher initial inoculum and changes in growth media. (**B**) Drop plate assay quantifying the apoplastic bacterial load following surface sterilization using two different compounds, ethanol and bleach. No significant difference in quantified bacterial load was observed. Data are presented as mean ± SD (n=5 independent biological replicates). Statistical differences were calculated using two-sample t-test.


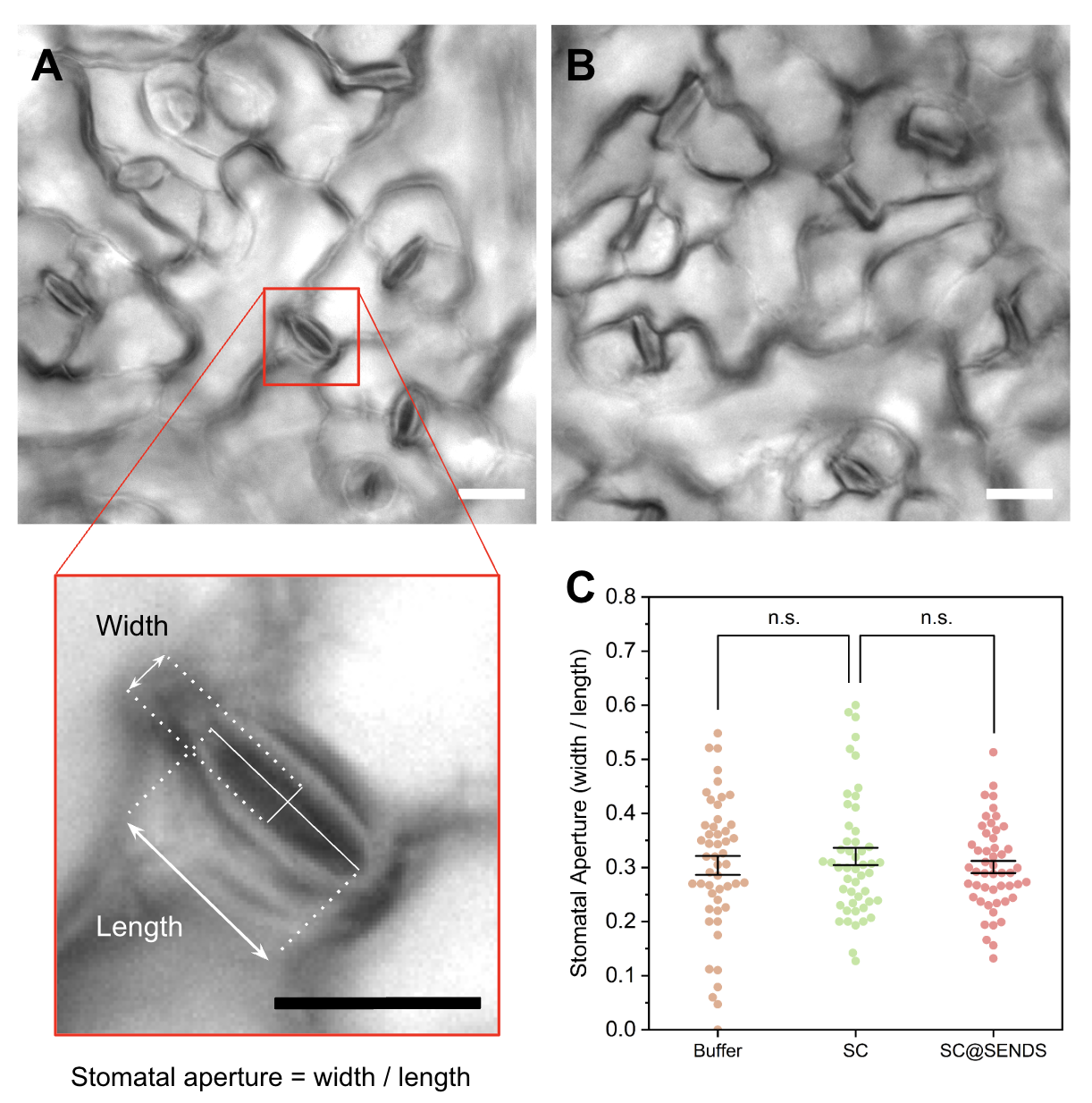


**Fig. S15. Stomatal aperture measurements.** Micrographs depicting stomata of leaves treated with (**A**) buffer and (**B**) ABA, resulting in larger and smaller average stomatal apertures respectively. Scale bar, 20 µm. (**C**) Stomatal aperture measurements for SC and SC@SENDS treated leaves. Data are presented as mean ± SD (n=50 technical replicates). Statistical differences were calculated using ANOVA with Tukey’s post-hoc test. No significant difference was observed between the tested groups, confirming that SC and SC@SENDS did not induce stomatal closure.


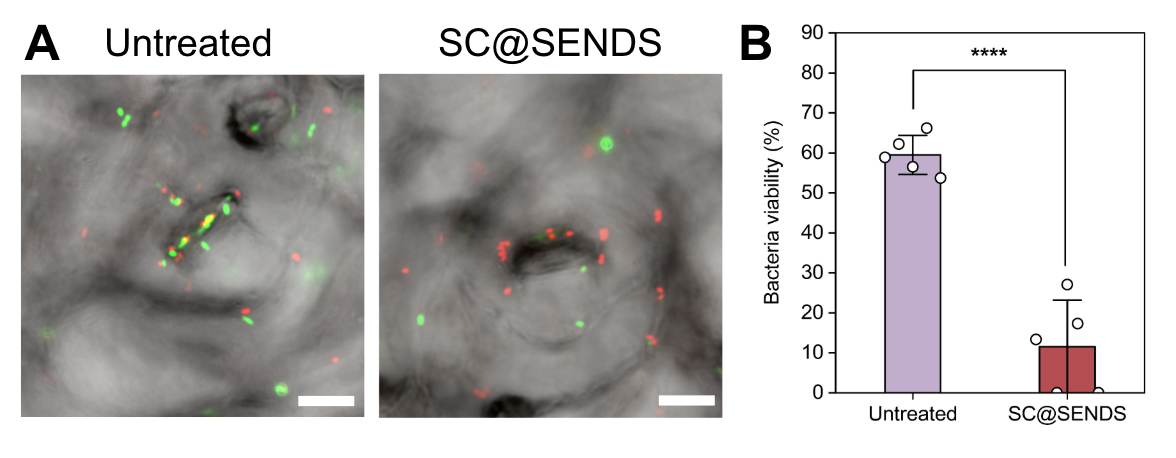


**Fig. S16. Visualizing the antibacterial activity of SC@SENDS.** (**A**) Additional confocal micrographs illustrating *X. campestris* entry to the stomata and the antimicrobial activity of SC@SENDS towards *X. campestris* surrounding the stomata. Scale bar, 10 µm. (**B**) Analysis of bacterial viability surrounding stomata based on confocal micrographs. Data are presented as mean ± SD (n=5 technical replicates). Statistical differences were calculated using two-sample t-test. ****P<0.0001.

**
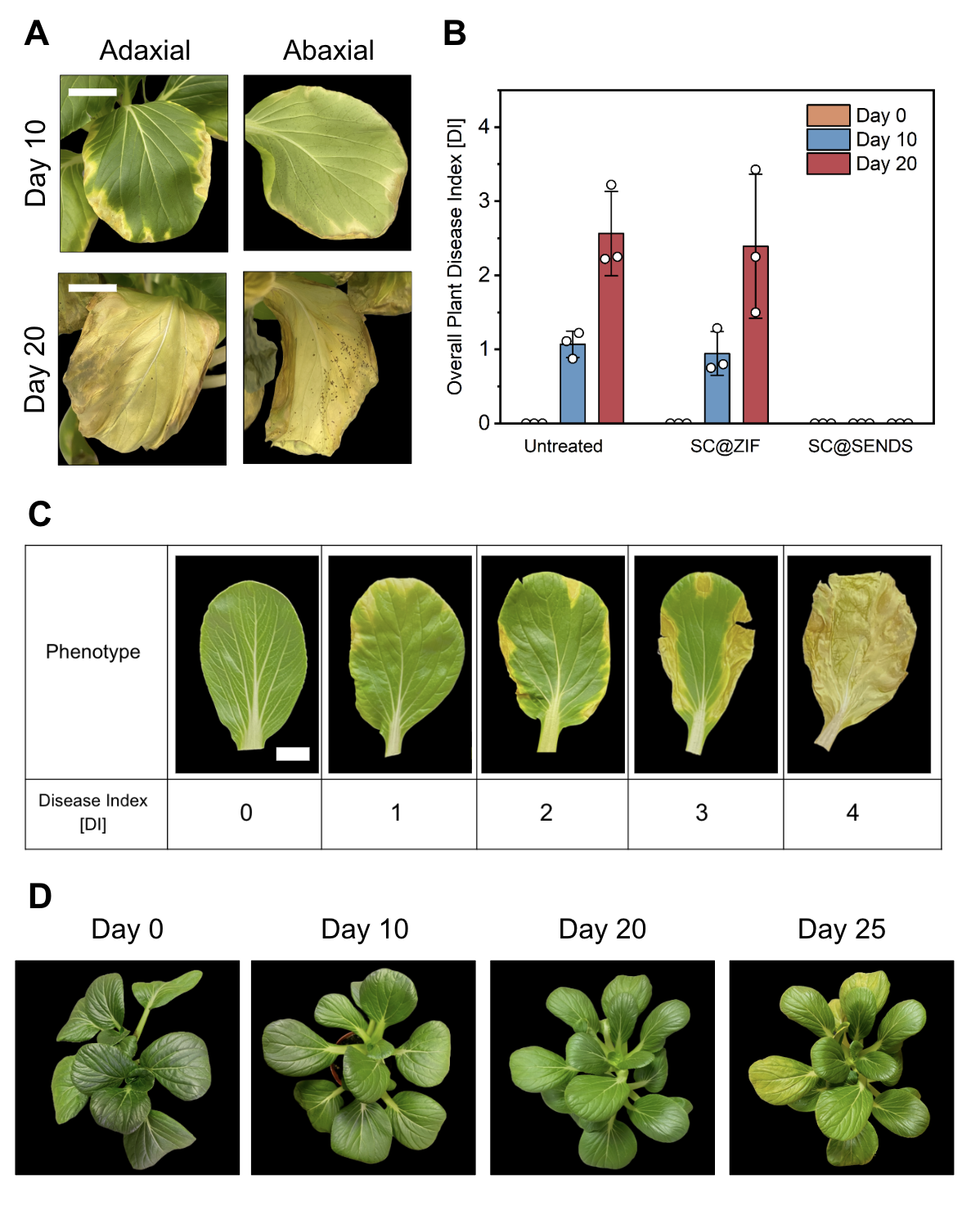
**

**Fig. S17. Phenotyping studies.** (**A**) Characteristic wedge-shaped chlorosis that expanded towards the midrib, resulting in fully necrotic leaves at 20 dpi. Black spots on the abaxial side increased in size and number with progression of the infection. (**B**) Evolution of overall plant disease index of tested plants across 20 days. (**C**) Disease Index (DI) scores - 0: no visible symptoms, 1: mild wedge-shaped chlorosis, 2: strong chlorosis, 3: strong chlorosis with onset of necrosis, 4: complete necrosis. Scale bar, 2 cm. (**D**) Phenotypic progression of uninoculated *B. chinensis* over 25 days, showing signs of chlorosis due to senescence of older leaves as the plant ages.


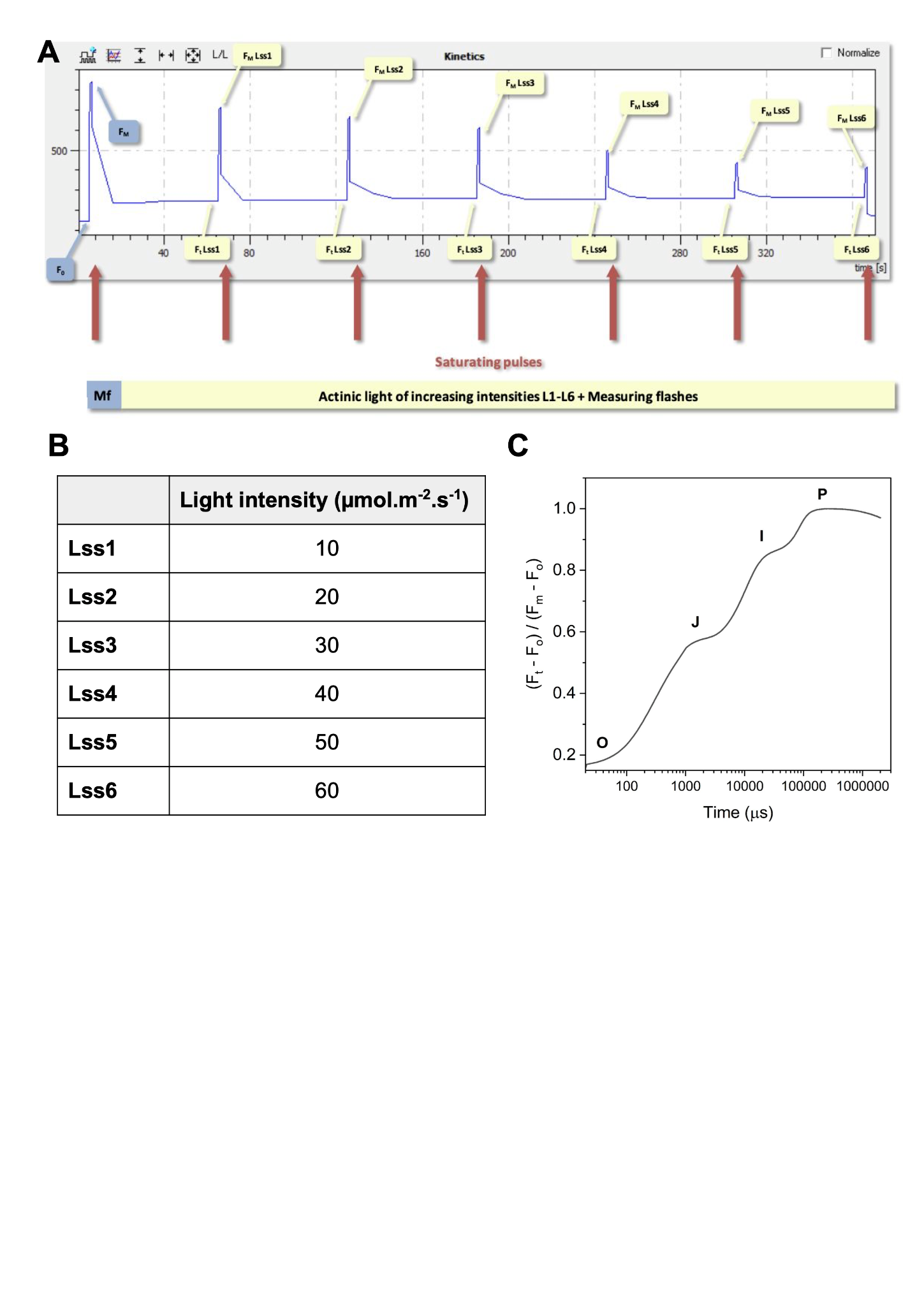


**Fig. S18.** **Chl-a measurement methods.** (**A**) Light Curve 1 protocol used to probe Chl-a fluorescence of plants. (**B**) Light intensity corresponding to each interval of actinic light used in Light Curve 1 protocol. (**C**) OJIP transient of healthy plant, illustrating distinct O, J, I and P steps.


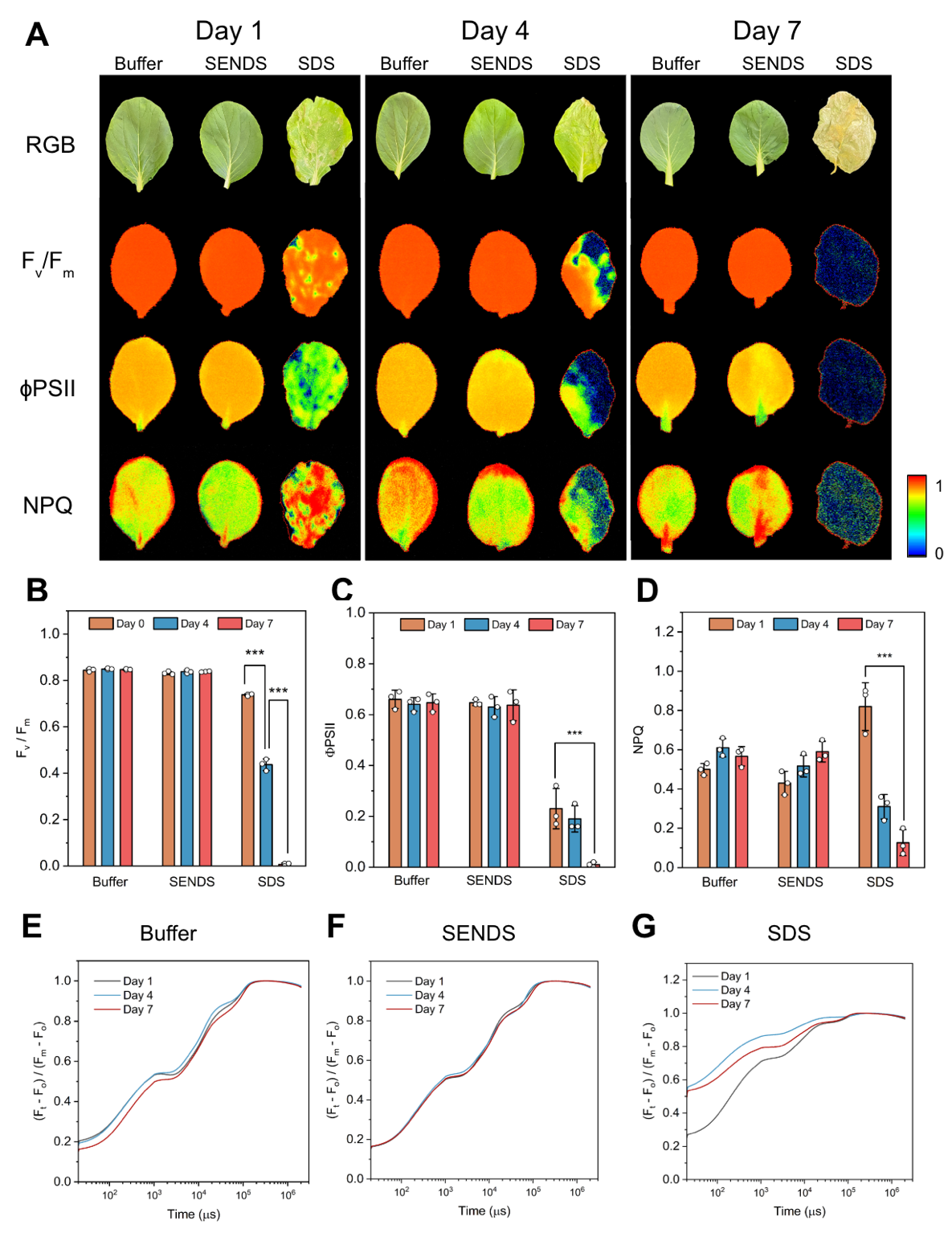


**Fig. S19.** **Chl-a measurements for biosafety tests.** (**A**) False color maps of sampled leaves from treated plants illustrating the distribution of Fv/Fm, ΦPSII and NPQ values across the surface of the leaves. Evolution of averaged (**B**) Fv/Fm, (**C**) ΦPSII and (**D**) NPQ of sampled leaves across the seven-day experimental duration. Data are presented as mean ± SD (n=3 independent biological replicates). Statistical differences were calculated using two-sample t-test. ***P<0.001. Evolution of OJIP transients in (**E**) buffer, (**F**) SENDS and (**G**) SDS-treated samples.


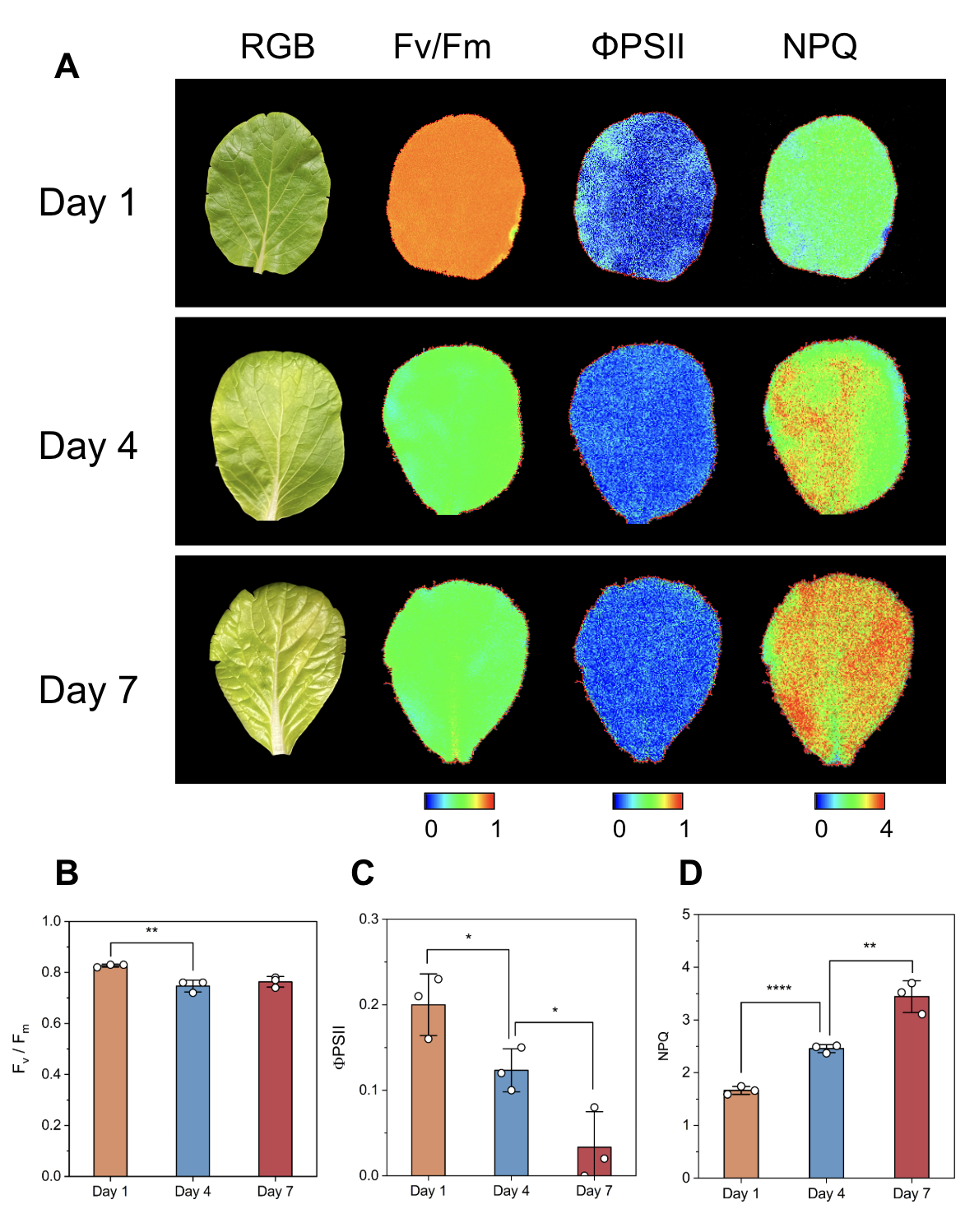


**Fig. S20**. **Chl-a measurements for plants treated with 0.5% SDS** (**A**) False color maps of sampled leaves from treated plants illustrating the distribution of Fv/Fm, ΦPSII and NPQ values across the surface of the leaves. Evolution of averaged (**B**) Fv/Fm, (**C**) ΦPSII and (**D**) NPQ of sampled leaves across the seven-day experimental duration. Data are presented as mean ± SD (n=3 independent biological replicates). Statistical differences were calculated using two-sample t-test. ****P<0.0001. **P<0.01, *P<0.05.
