# Supplementary material for: Stomata-targeted nanocarriers enhance plant defense against pathogen colonization
Source: Nat Commun. 2025 May 23;16:4816. doi: 10.1038/s41467-025-60112-w (PMC12102249; doi:10.1038/s41467-025-60112-w)
Supplement: Supplementary file 2 — Reporting Summary [file 41467_2025_60112_MOESM2_ESM.pdf]

nature portfolio

Corresponding author(s): Tedrick Thomas Salim Lew

Last updated by author(s): May 5, 2025

Reporting Summary

Nature Portfolio wishes to improve the reproducibility of the work that we publish. This form provides structure for consistency and transparency in reporting. For further information on Nature Portfolio policies, see our Editorial Policies and the Editorial Policy Checklist.

Statistics

For all statistical analyses, confirm that the following items are present in the figure legend, table legend, main text, or Methods section.

n/a	Confirmed	
☐	☒	The exact sample size (*n*) for each experimental group/condition, given as a discrete number and unit of measurement
☐	☒	A statement on whether measurements were taken from distinct samples or whether the same sample was measured repeatedly
☐	☒	The statistical test(s) used AND whether they are one- or two-sided<br>*Only common tests should be described solely by name; describe more complex techniques in the Methods section.*
☐	☒	A description of all covariates tested
☒	☐	A description of any assumptions or corrections, such as tests of normality and adjustment for multiple comparisons
☐	☒	A full description of the statistical parameters including central tendency (e.g. means) or other basic estimates (e.g. regression coefficient) AND variation (e.g. standard deviation) or associated estimates of uncertainty (e.g. confidence intervals)
☐	☒	For null hypothesis testing, the test statistic (e.g. *F*, *t*, *r*) with confidence intervals, effect sizes, degrees of freedom and *P* value noted<br>*Give P values as exact values whenever suitable.*
☒	☐	For Bayesian analysis, information on the choice of priors and Markov chain Monte Carlo settings
☒	☐	For hierarchical and complex designs, identification of the appropriate level for tests and full reporting of outcomes
☒	☐	Estimates of effect sizes (e.g. Cohen's *d*, Pearson's *r*), indicating how they were calculated

Our web collection on statistics for biologists contains articles on many of the points above.

Software and code

Policy information about availability of computer code

Data collection	Zetasizer Nano, BioTek Gen5, JEOL PC-SEM, Oxford Instruments INCA, JEOL TEM Controller Application, Emsis RADIUS, Bruker NanoScope, Olympus FV31S, PSI FluorCam10, PSI FluorPen
Data analysis	ImageJ (version 1.54f), Origin Lab (version 2021b)

For manuscripts utilizing custom algorithms or software that are central to the research but not yet described in published literature, software must be made available to editors and reviewers. We strongly encourage code deposition in a community repository (e.g. GitHub). See the Nature Portfolio guidelines for submitting code & software for further information.

Data

Policy information about availability of data

All manuscripts must include a data availability statement. This statement should provide the following information, where applicable:

- Accession codes, unique identifiers, or web links for publicly available datasets
- A description of any restrictions on data availability
- For clinical datasets or third party data, please ensure that the statement adheres to our policy

All data supporting the findings in the paper are present in the main manuscript and the Supplementary Information. Source data for Figs. 2C-F, 3B, 4B, 5B-C, 6B-G, 7A-C and Supplementary Figs. S1A, D-F, S2A-B, S4D, S5C-E, S9, S10B-C, S11A-B, S12C-D, S13B, E, S14B, S15C, S16B, S17B, S19B-G, S20B-D are provided in the source data file. Any raw data can be obtained upon request to the corresponding author.

Research involving human participants, their data, or biological material

Policy information about studies with human participants or human data. See also policy information about sex, gender (identity/presentation), and sexual orientation and race, ethnicity and racism.

Reporting on sex and gender	N.A.
Reporting on race, ethnicity, or other socially relevant groupings	N.A.
Population characteristics	N.A.
Recruitment	N.A.
Ethics oversight	N.A.

Note that full information on the approval of the study protocol must also be provided in the manuscript.

Field-specific reporting

Please select the one below that is the best fit for your research. If you are not sure, read the appropriate sections before making your selection.

- [x] Life sciences
- [ ] Behavioural & social sciences
- [ ] Ecological, evolutionary & environmental sciences

For a reference copy of the document with all sections, see nature.com/documents/nr-reporting-summary-flat.pdf

Life sciences study design

All studies must disclose on these points even when the disclosure is negative.

Sample size	The sample size for plant studies is at least 3-5 biological replicates for the experiments detailed in the manuscript. Samples were chosen based on the criteria detailed in the Methods section.
Data exclusions	No data was excluded.
Replication	All replication attempts were successful.
Randomization	Plants were chosen randomly according to the Methods section. The selection of leaf area for confocal imaging was also detailed in the manuscript.
Blinding	N.A.

Reporting for specific materials, systems and methods

We require information from authors about some types of materials, experimental systems and methods used in many studies. Here, indicate whether each material, system or method listed is relevant to your study. If you are not sure if a list item applies to your research, read the appropriate section before selecting a response.

Materials & experimental systems

n/a	Involved in the study	
☐	☒	Antibodies
☒	☐	Eukaryotic cell lines
☒	☐	Palaeontology and archaeology
☒	☐	Animals and other organisms
☒	☐	Clinical data
☒	☐	Dual use research of concern
☐	☒	Plants

Methods

n/a	Involved in the study	
☒	☐	ChIP-seq
☒	☐	Flow cytometry
☒	☐	MRI-based neuroimaging

Antibodies

Antibodies used	Anti-Pectic polysaccharide (alpha-1,5-arabinan) [LM6] Antibody (Kerafast ELD008) LOT: 211220<br>Cy3 AffiniPure Goat Anti-Rat IgG (Fab') fragment specific (Jackson ImmunoResearch 112-165-006) LOT: 163872<br>Fluorescein (FITC) AffiniPure Goat Anti-Rat IgG, Fc fragment specific (Jackson ImmunoResearch, 112-095-008) LOT: 159572<br>His-Tag (H-3) Antibody (Santa Cruz Biotechnology Inc. sc-8036) LOT: D1823

Validation	N.A.

Dual use research of concern

Policy information about dual use research of concern

Hazards

Could the accidental, deliberate or reckless misuse of agents or technologies generated in the work, or the application of information presented in the manuscript, pose a threat to:

No	Yes	
☒	☐	Public health
☒	☐	National security
☒	☐	Crops and/or livestock
☒	☐	Ecosystems
☒	☐	Any other significant area

Experiments of concern

Does the work involve any of these experiments of concern:

No	Yes	
☒	☐	Demonstrate how to render a vaccine ineffective
☒	☐	Confer resistance to therapeutically useful antibiotics or antiviral agents
☒	☐	Enhance the virulence of a pathogen or render a nonpathogen virulent
☒	☐	Increase transmissibility of a pathogen
☒	☐	Alter the host range of a pathogen
☒	☐	Enable evasion of diagnostic/detection modalities
☒	☐	Enable the weaponization of a biological agent or toxin
☒	☐	Any other potentially harmful combination of experiments and agents

Plants

Seed stocks	B. chinensis, V. faba, O. sativa and H. vulgare seeds were procured commercially from The Living Centre and Horti Flora. Wild-type A. thaliana seeds were obtained from ABRC. mCherry-expressing A. thaliana mutant seeds were described previously (Lau et al. (2014), Science, 345, 1605-1609)
Novel plant genotypes	N.A.
Authentication	N.A.
